# Index sorting resolves heterogeneous murine hematopoietic stem cell populations

**DOI:** 10.1016/j.exphem.2015.05.006

**Published:** 2015-09

**Authors:** Reiner Schulte, Nicola K. Wilson, Janine C.M. Prick, Chiara Cossetti, Michal K. Maj, Berthold Gottgens, David G. Kent

**Affiliations:** a Cambridge Institute for Medical Research, University of Cambridge, Cambridge, United Kingdom; b Department of Haematology, University of Cambridge, Cambridge, United Kingdom; c Wellcome Trust/MRC Stem Cell Institute, University of Cambridge, Cambridge, United Kingdom

## Abstract

Recent advances in the cellular and molecular biology of single stem cells have uncovered significant heterogeneity in the functional properties of stem cell populations. This has prompted the development of approaches to study single cells in isolation, often performed using multiparameter flow cytometry. However, many stem cell populations are too rare to test all possible cell surface marker combinations, and virtually nothing is known about functional differences associated with varying intensities of such markers. Here we describe the use of index sorting for further resolution of the flow cytometric isolation of single murine hematopoietic stem cells (HSCs). Specifically, we associate single-cell functional assay outcomes with distinct cell surface marker expression intensities. High levels of both CD150 and EPCR associate with delayed kinetics of cell division and low levels of differentiation. Moreover, cells that do not form single HSC-derived clones appear in the 7AAD^dim^ fraction, suggesting that even low levels of 7AAD staining are indicative of less healthy cell populations. These data indicate that when used in combination with single-cell functional assays, index sorting is a powerful tool for refining cell isolation strategies. This approach can be broadly applied to other single-cell systems, both to improve isolation and to acquire additional cell surface marker information.

## Introduction

Heterogeneity in cell populations poses a significant challenge to understanding the biology of normal and malignant single cells [1]. Advanced multiparameter cell sorting has enabled the isolation of rare subpopulations with properties distinct from those of bulk cell populations, but the vast majority of such populations remain at purities less than 50%, with many fractions substantially lower. This means that when cells are studied at a single-cell level for expression of genes or proteins or are assessed for their functional activity, the majority of the cells assessed are not actually the cells of interest. Therefore, techniques are required either to obtain near-pure cell fractions or to associate individual cells with multiple individual outcomes. The latter is particularly complicated because the majority of such techniques (e.g., gene expression) destroy the cell of interest, making it impossible to assess in a functional assay.

Stem cells are generally rare cell populations, and cell number is typically limited in adult mammalian systems [2], often yielding just a few hundred cells in a single experiment. For example, functional mouse blood stem cells are present at a frequency of ∼0.004% in the bone marrow and orders of magnitude less in the peripheral blood [3]. Performing large numbers of functional screens using different combinations of multiple cell surface markers is virtually impossible because stem cell transplantation is required to validate stem cell function. Efforts have therefore been restricted to adding or subtracting one marker at a time [4], and virtually no studies have assessed the impact of different levels of expression across multiple markers.

Single-cell sorting is a powerful tool in biomedical research as it allows separation and analysis of individual cells. New instrument developments have improved the index sorting function of several commercial cell sorters, making it possible to review the complete flow phenotype of every single cell sorted into a 96-or 384-well plate [5,6]. This technique has already been used to analyze gene expression in planarian stem cells [7] and the diversity of antibody repertoires in a high-throughput manner [5,6], and most recently we have reported its application to stem cell populations [8].

Here we report the use of index sorting in rare mouse hematopoietic stem cell populations as a method to survey multiple different combinations of cell surface marker intensities to resolve subpopulations in cell fractions and to improve purities of functional outcomes. By linking functional in vitro readouts that associate with stem cell activity to individual single-cell surface marker profiles, we are able to identify contaminating nonfunctional cell fractions and determine the functional importance of higher or lower levels of the stem cell markers EPCR and CD150.

## Methods

### BD Influx setup and preparation of plate holder

All cell sorting experiments were performed on a BD Influx cell sorter running BD FACS Sortware. Laser alignment was performed using eight-peak rainbow beads (Spherotech), and drop delay was determined using BD Accudrop beads.

**Figure 1:**
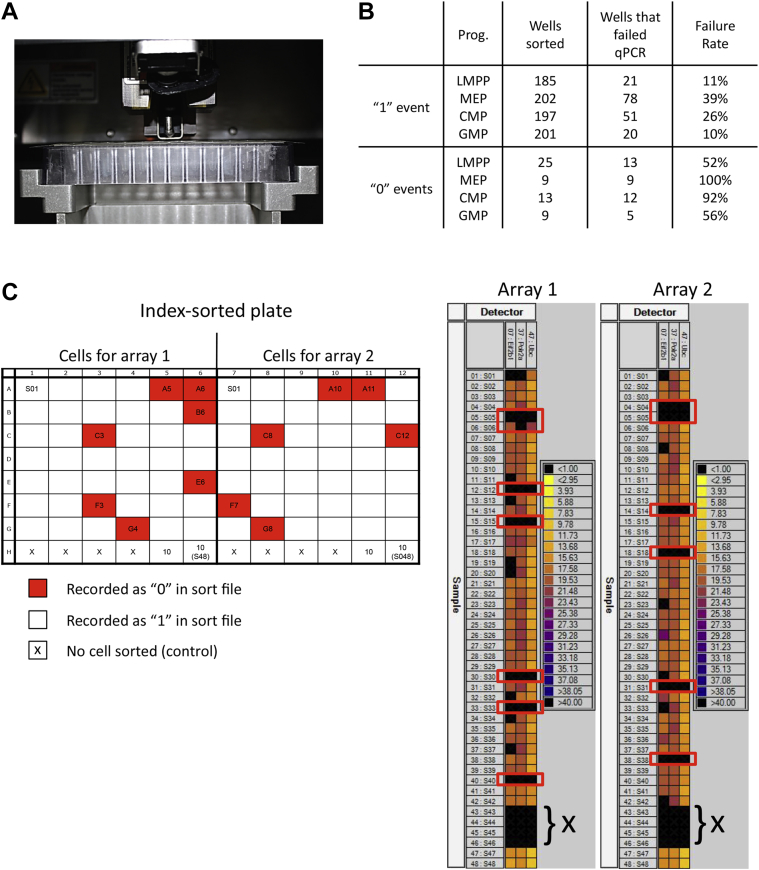
Modification of BD Influx 96-well plate holder and workflow of index sorting and analysis. (**A**) Inserting the PCR plate into a 96-well polycarbonate rack and securing it with pressure-sensitive adhesive resulted in a flat PCR plate and a short distance between breakoff point and plate surface. (**B**) Summary statistics of wells where either ‘1’ event or ‘0’event was indicated by the software, and their subsequent success or failure in single-cell qPCR assays. Notably, the average failure rate of wells that the software indicated as empty was 75%, compared with only 22% for those wells indicated as having ‘1’ cell. The latter failure rate is reflective of the technical limitations of the single-cell qPCR assay. (**C**) Representative experiment in which individual wells of a 96-well plate were assessed as having ‘0'or ‘1’ cell present by the Sortware software. The 96-well plate was run in two independent microfluidic chips, whereas the contents of each of 48 wells were run in a single-cell PCR; the results for three housekeeping genes are illustrated (Eif2b1, Polr2a, and Ubc). Notably the wells that had ‘0’ event sorted in these two arrays also did not show signal in the PCR.

The plate holder apparatus on a BD Influx does not hold a nonskirted 96-well PCR plate tightly. To create a fitting holder, a 96-well polycarbonate rack typically used to hold individual 1.4-mL polypropylene round-bottom tubes was used. By removing the legs of the rack and shaving the bottom surface to be flat, we were able to create a rigid fit in the sort tray of the Influx sorter. Standard 96-well PCR plates were able to fit easily into the rack and were secured using individual portions of a pressure-sensitive adhesive (e.g., Blu-Tack) in several locations within the rack. To establish the alignment of the sort plate on the sort stage we performed sorts of 10 beads onto the lid of a 96-well plate. Cells were then index sorted into wells of a 96-well plate and analyzed further. To determine the precision of the cell sorter, cells were index sorted into 96-well PCR plates to perform Fluidigm real-time PCR analysis (Fig. 1C).

### Export of index sort files

For each experiment, index sort files were exported using BD FACS Sortware. These files were analyzed to assess the rare events in which no cell had been sorted (Fig. 1C). These wells were then manually entered into the Sortware index sort layout, and cells were sorted into these cells. A second index sort file was exported afterward. For all experiments, fcs-files and CSV-files were exported for comparison with the index sort files using Microsoft Excel and R.

### Purification of HSCs and progenitors

Bone marrow cells were collected from the femurs, tibias, and iliac crests of 8- to 16-week-old C57BL/6 mice and were depleted of red blood cells in an ammonium chloride lysis step (STEMCELL Technologies). HSCs and progenitors were isolated using the following antibodies: CD45-FITC (Clone 30-F11 Biolegend); EPCR-PE (Clone RMEPCR1560, STEMCELL); CD150-PE-Cy7 (Clone TC15-12F12.2, Biolegend); CD48-APC (Clone HM48-1, Biolegend); Sca-1-Pacific Blue (Clone E13-161.7, Biolegend); FLT3-PE or PE-Cy5 (Clone A2F10, eBioscience); CD34-FITC (Clone RAM34, BD Biosciences); c-kit APC-Cy7 (Clone 2B8, Biolegend); and a panel of lineage markers (Hematopoietic Progenitor Enrichment Cocktail, STEMCELL) plus streptavidin-V500 (BD Biosciences). Progenitors were isolated as follows: 197 common myeloid progenitors (Lin^−^c-kit^+^Sca-1^−^CD34^+^FcγR^low^, CMPs); 202 megkaryocyte–erythrocyte progenitors (Lin^−^c-kit^+^Sca-1^−^CD34^−^FcγR^low^, MEPs); 201 granulocyte–macrophage progenitors (Lin^−^c-kit^+^Sca-1^−^CD34^+^FcγR^hi^, GMPs); and 185 lymphoid-primed multipotent progenitors (Lin^−^c-kit^+^Sca-1^+^CD34^+^Flt3^+^, LMPPs). Cells were sorted using a BD Influx sorter equipped with 355-, 405-, 488-, 561-, and 640-nm lasers. For single-cell gene expression assays, cells were sorted into individual wells of 96-well PCR plates using the modified plate holder illustrated in Figure 1. For single-cell in vitro assays, cells were sorted into individual wells of 96-well tissue culture plates. All mice were kept under specific pathogen-free conditions, and all procedures were performed according to UK Home Office regulations.

### Single-HSC short-term cultures

EPCR^++^CD150^+^CD48^−^CD45^+^ (E-SLAM) HSCs were sorted and cultured in STEMSPAN medium containing 300 ng/mL stem cell factor, 20 ng/mL interleukin-11, glutamine, penicillin and streptomycin, β-mercaptoethanol, and 10% fetal calf serum, as described previously [12,23]. After 24 hours, wells were scored for the presence of a single cell and counted each day to track the clonal growth of individual cells. For the immunophenotyping studies, clones were individually stained and assessed for the expression of Sca-1, c-Kit, and a panel of lineage markers along with 7-AAD (Invitrogen) to mark dead cells.

### Clone size calculations and antibody information for in vitro cultures

Clone size calculations were determined as previously described [12]. After 10 days of culture, single HSC-derived clones were estimated to be small (50–5,000 cells), medium (5,000–20,000 cells), or large (≥20,000). No clones had fewer than 50 cells. Ten-day clones were stained with biotinylated lineage marker antibodies (Haematopoietic Progenitor Enrichment Cocktail, STEMCELL), c-Kit-APC (BD) and Sca-1-Pacific Blue (Biolegend). To enumerate cells, a defined number of fluorescent beads (Trucount Control Beads, BD) were added to each well, and each sample was backcalculated to the proportion of the total that were run through the cytometer. Small clones were not able to be assessed individually by flow cytometry and were pooled; the percentage of c-Kit^+^Sca^+^Lin^−^ (KSL) cells was greater than 95%. Flow cytometry was performed on an LSRII Fortessa (BD), and all data were analyzed using Flowjo 10.0.7 (Treestar, USA).

### Single-cell cobblestone area-forming cell assay

The CAFC assay was modified from de Haan et al. [24] and performed as described previously [17]. The FBMD stromal cell line (obtained from G. de Haan, originally from R. Ploemacher, Erasmus University, Rotterdam, Netherlands) was maintained in Quantum 333 complete fibroblast medium with L-glutamine (PAA Laboratories) plus β-mercaptoethanol, penicillin, and streptomycin. Stromal cell layers were established in the inner 60 wells of 96-well plates and were incubated at 33°C. Prior to sorting, medium was replaced with 200 μL Iscove's modified Dulbecco's medium, 20% horse serum, 10^−5^ mol/L hydrocortisone, 10^−4^ mol/L β-mercaptoethanol plus penicillin and streptomycin (collectively, CAFC medium). Each well was seeded with a single HSC, and wells were examined each week for 12 weeks for the presence or absence of cobblestone areas (identified as colonies of at least five flat nonrefractile cells growing underneath the stromal layer). Medium was changed each week by removing most of the old medium using a multichannel pipette (without disturbing the stromal layer) and adding 180 μL of fresh CAFC medium.

### Single-cell gene expression analysis

Single-cell gene expression analysis was performed as described previously [9]. Briefly, single cells were sorted directly into individual wells of 96-well plates containing lysis and pre-amplification mix. Reverse transcription and specific target amplification were performed in the same plates 24 hours after sorting. cDNA was diluted 1:5 with TE before qPCR on the BioMark HD. For the qPCR, Taqman assays (Life Technologies) and cDNA samples were loaded into a 48.48 Dynamic Array (Fluidigm) and then transferred to the BioMark HD for qPCR. A successful single-cell qPCR assay was determined by successful amplification of 3 control genes in addition to at least 50% of the 45 additional genes assessed in each microfluidic chip.

### Statistical analysis

Student's *t*-test was used to analyze differences in the average numbers of cells, progenitor cells, and mature cells per clone. Pearson correlation analysis was performed for CD150 and EPCR for Figure 3C. All statistical analyses were performed using GraphPad Prism Version 5.

## Results

### Modified sort tray apparatus helps to stabilize PCR plates

Single-cell polymerase chain reaction (PCR) poses numerous technical challenge, and the most published protocols require cells to be sorted into very small volumes of liquid, often less than 1–2 μL [9–11]. It was therefore important to have plates in close proximity to the deflection point of the single cell and a rigid sort plate setup that does not move during the sort. Standard PCR plates are not rigid, and skirted PCR plates are not usable in many standard PCR machines. Therefore, we developed a plate-holding apparatus for the BD Influx sorter (BD Biosciences) using a 96-well polycarbonate rack typically used to hold individual 1.4-mL polypropylene round-bottom tubes (see Methods and Fig. 1A).

### Index sorting used as a tool for determining transcriptional differences in single stem cells

To develop a tool to understand the molecular heterogeneity within blood stem cell populations, we took advantage of the index-sort mode on the BD Influx sorter. This feature captures the individual fluorescence data for each parameter for each single cell collected. Cells were sorted into 96-well PCR plates, and individual parameter information was collected in a spreadsheet that could be directly correlated to the same well in the PCR plate, similar to the method employed by Hayashi et al. [7]. After index sorting of single cells into a 96-well PCR plate, the sort statistic was exported as .txt file using BD Sortware software. The file was opened using Microsoft Notepad, and wells were analyzed for individual parameters. In some cases, the file indicated that no event had been sorted (Fig. 1B). Wells identified as empty by the software were significantly more likely to fail the single-cell quantitative PCR (qPCR) protocol (Fig. 1B, C) (*p* = 0.014). In total, we assessed 841 hematopoietic progenitor cells in this manner (see Methods for progenitor isolation strategies), and the software indicated a total of 56 empty wells, 75% of which failed to show signal in the qPCR protocol. The failure rate of the single-cell qPCR assay when a “1” was indicated by the software was 22% (Fig. 1B). Together these data suggest that in the majority of cases, a “0” meant that no cell was actually sorted into the well. For subsequent experiments, therefore, we refined our approach to check immediately postsort for empty wells (as defined by the software) and re-sorted single events into those wells. This refinement enabled all downstream hematopoietic stem cell (HSC) assays to be run more efficiently, resulting in substantial savings in reagent costs.

### Index sorting helps to resolve viability gates

**Figure 2:**
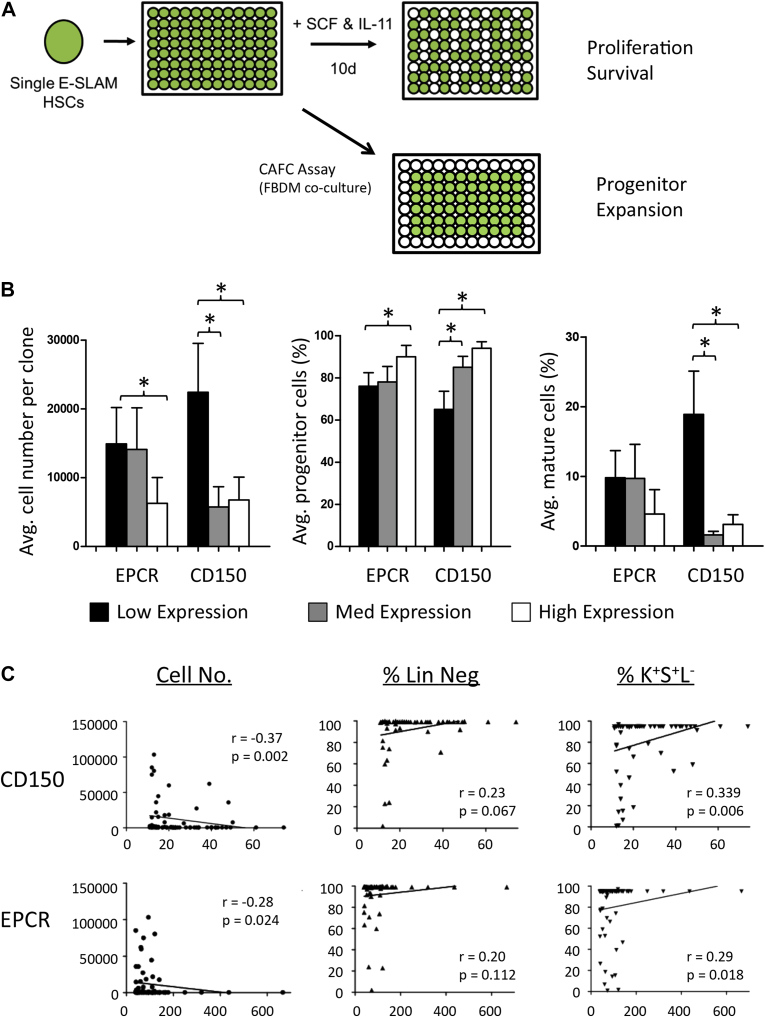
Index sorting permits interrogation of the complete range of cell surface marker expression. (**A**) Single HSCs were sorted into short- and long-term in vitro assays to determine their functional capacity. Cells were index sorted, and colony outcomes were retrospectively assessed across the dynamic range of CD150 and EPCR expression (two positive markers for HSCs). (**B**) Expression levels within the positive fractions of EPCR and CD150 were divided into three fractions (low expression, medium expression, and high expression) based on their individual mean fluorescence intensity in each channel. Left: Average number of cells created per clone. Middle: Percentage of progenitor cells. Right: Number of mature cells produced. Higher expression of both CD150 and EPCR is associated with smaller, less differentiated cells. (**C**) Correlation analysis of individual markers. CD150 and EPCR are significantly correlated with cell number (*p* = 0.002 and *p* = 0.024) and progenitor content (*p* = 0.006 and *p* = 0.018). *IL-11* = interleukin 11; *SCF* = stem cell factor. p<0.05.

Highly purified EPCR^++^CD150^+^CD48^−^CD45^+^ (E-SLAM) HSCs have previously been found to be highly efficient (>90%) at making colonies under supportive cell culture conditions when a strict viability gate is set [12,13]. When flow-sorting data were analyzed, two populations were identified that would typically be excluded by a strict viability gate: a low forward scatter (FSC) population and a 7-AAD^dim^ (7-AAD = 7-aminoactinomycin D) population (Fig. 2A). To test whether these two populations possessed colony-forming potential, we sorted on a permissive viability gate and used the index sorting data to retrospectively assess colony-forming potential. Displaying these populations using the CD45 and EPCR markers indicates that the low FSC population clusters with the majority of cells, but the 7-AAD^dim^ population had a large cluster of cells in the bottom left corner of the standard CD45/EPCR double-positive gate (Fig. 2A). Backgating predicted that higher than average 7-AAD staining would be associated with events that could not form clones, despite all events falling in a typical 7-AAD-negative gate. Indeed, when a more restrictive 7-AAD gate was set, the cluster in the bottom left corner was no longer present (Fig. 2B), and this finding was confirmed by in vitro functional data that indicated HSCs unable to form colonies had higher levels of 7-AAD at the time of sorting. In a representative experiment illustrated in Figure 2C, cells that did not form clones tended to have higher 7-AAD fluorescence intensity.

### Index sorting permits high-throughput resolution of sorting gates

**Figure 3:**
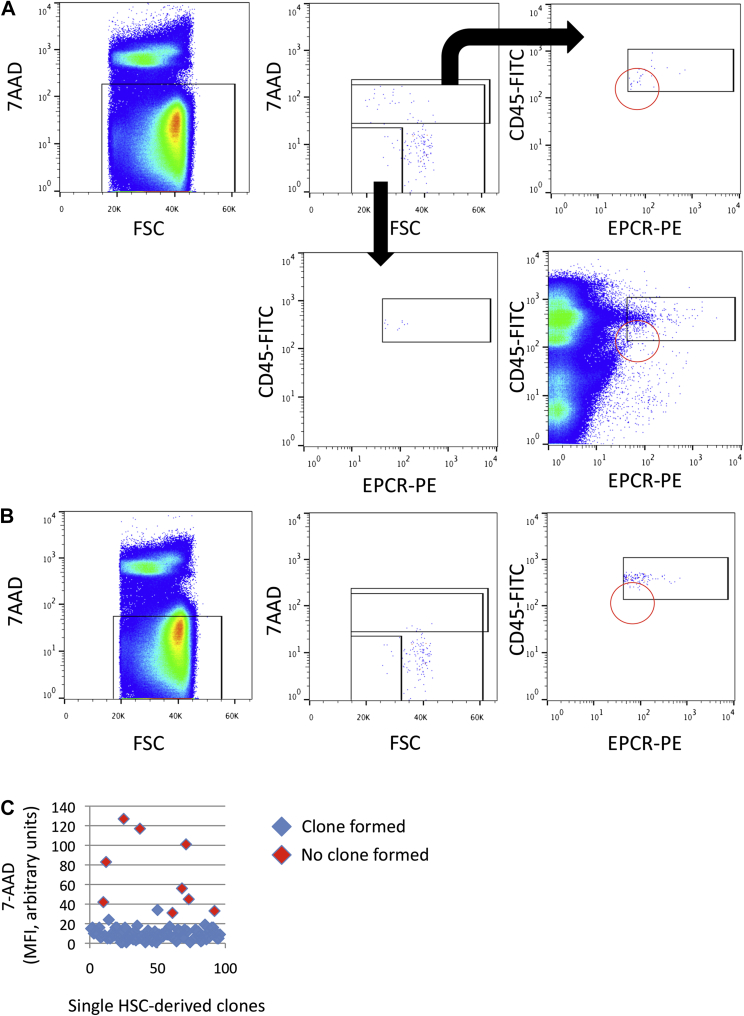
Index sorting helps refine cell populations with growth potential. (**A**) Mouse bone marrow cells were stained with cell surface markers (EPCR, CD150, CD48, and CD45) to identify viable (7-AAD-negative) hematopoietic stem cells. When HSCs were backgated on the FSC/7-AAD plot, two populations separated from the main cluster: a low FSC population and a low 7-AAD-positive population. Both populations were fractionated and displayed on an EPCR/CD45 plot, which showed that compared with the low FSC and main population cells, those expressing small amounts of 7-AAD clustered in the bottom left corner of the plot. (**B**) A more restrictive 7-AAD gate was applied. Very few cells were observed in the bottom left corner. (**C**) Index-sorted cells were placed into single-cell in vitro progenitor assays, and those expressing high amounts of 7-AAD did not grow colonies (left panel). Notably, only three of these nine cells would fall within the *red circle* indicated in (**A**) and (**B**).

Typically, researchers attempting to further refine gates sort independent populations of cells expressing high, low, or no levels of markers, which makes large numbers of tests difficult when dealing with rare populations (e.g., HSCs represent approximately 0.004% of mouse bone marrow cells, and only 800–1,200 are obtained from a typical mouse bone marrow) [4,14]. Index sorting permitted us to take a standard HSC sorting and retrospectively assess the impact of different levels of HSC markers in both short-term and long-term in vitro assays (Fig. 3A).

Hematopoietic stem cells have been found to be heterogeneous in their function, separating into distinct classes based on their self-renewal durability and the number and type of mature cells they produce [3,15]. The E-SLAM population contains approximately 40% serially transplantable multilineage HSCs, 20% finite self-renewal HSCs (cannot serially transplant), and 40% cells that do not read out at all in transplantation assay [13]. Previous studies have also reported that HSCs are slow to enter cell cycle [15,16], express fewer markers of differentiation in short-term cultures containing stem cell factor and interleukin-11 [12], and appear later in long-term in vitro assays [17] (e.g., the cobblestone area-forming cell [CAFC] assay). EPCR and CD150 are positive markers of long-term HSCs and have a broad range of expression, so we asked whether index sort data could help resolve the population further than previous studies [4,13,18].

**Figure 4:**
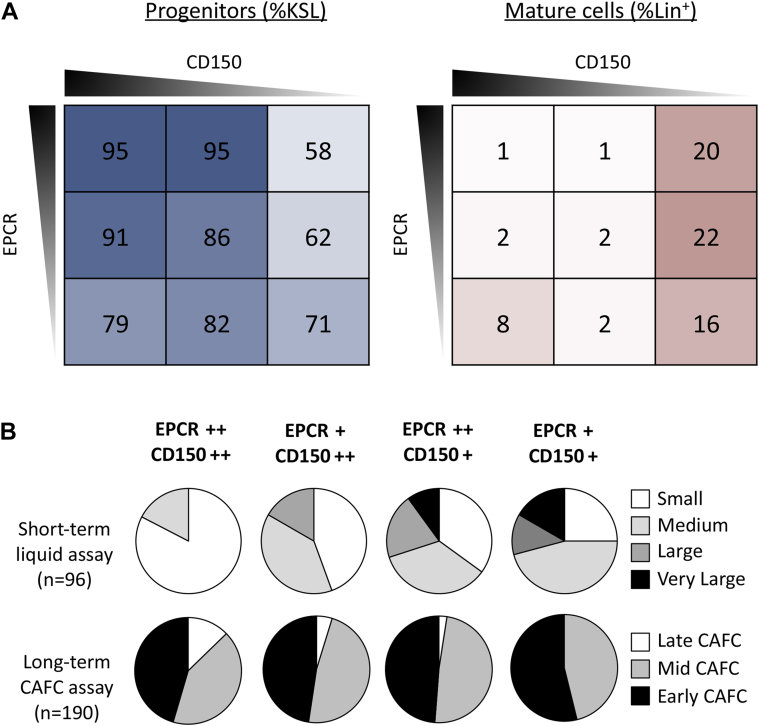
CD150 and EPCR intensity can be used to isolate less differentiated, slow-dividing HSCs. (**A**) Percentages of progenitor cells (KSL, left panel) and mature lineage marker-positive cells (right panel) are displayed for the various combinations of CD150 (high, medium, and low categories from left to right) and EPCR (high, medium, and low categories from top to bottom). (**B**) Functional assays indicate that high levels of EPCR and CD150 associate with short- and long-term in vitro activity. Small clone size under 10-day culture conditions and delayed appearance of colonies in the CAFC assay have previously been associated with HSC retention. The top 50% of CD150 (CD150^++^) and EPCR (EPCR^++^) expression was the most effective combination for promoting clones with HSC properties (late CAFCs and small clones).

First we assessed each marker individually for its impact on cell proliferation and differentiation using both categorical (Fig. 3B) and correlation analyses (Fig. 3C). For the categorical analysis, each population was divided into low expressing cells (bottom third of fluorescence), medium expressing cells (middle third), and high expressing cells (top third). As individual markers, both CD150 expression and EPCR expression positively associated with stem cell characteristics in short-term cultures (smaller average cell number, higher proportion of progenitor cells, and lower proportion of mature cells, n = 64 single cell-derived clones) (Fig. 3B). Whereas only EPCR^high^-expressing cells had these properties, both the CD150^medium^- and CD150^high^-expressing cells possessed these properties. When analyzed in combination, it is apparent that CD150^low^ is the primary driver of differentiation (Fig. 4A).

Next, we assessed the combination of both markers with respect to their impact on clone size and the timing of CAFC appearance. Long-term HSCs have previously been found to give rise to small or medium-sized clones [12,16], and the late appearance of CAFCs has been previously associated with one of the HSC populations that has durable self-renewal [17]. We divided CD150 and EPCR expression separately into high expressers (top 50%) and low expressers (bottom 50%) and established four categories (EPCR^++^CD150^++^, EPCR^+^CD150^++^, EPCR^++^CD150^+^, and EPCR^+^CD150^+^). Compared with all other populations, cells that were EPCR^++^CD150^++^ were enriched for small and medium clones and also contained the highest proportion of late CAFCs, as indicated by the white sections of the pie graphs in Figure 4B.

Together these data indicate that in combination, high levels of CD150 and EPCR expression contain more HSCs with durable self-renewal potential compared with all other fractions. It further suggests that although some HSCs would be lost, setting stricter gates for CD150 and EPCR expression would enrich for more highly purified fractions. Sorting gate refinement using index sorting can be applied to any population that can be linked to a defined single-cell functional assay.

## Discussion

Adult stem cells are typically rare and not very numerous. Their functional assays are time consuming, expensive, and cumbersome, thereby limiting the number and types of assays that can be performed. Sophisticated approaches are therefore required to determine which function or property belongs to which single cell. Index sorting offers the unique opportunity to avoid prospective sorting of cells and enables questions to be asked retrospectively of multiple different sorting strategies. Our data indicate that index sorting can resolve flow-sorted populations and is particularly powerful when coupled with single-cell functional assays from rare cell populations.

The influence of different levels of expression within a standard gate is not well studied. Broad categorization of cells into low, medium, and high fractions can be useful for determining the impact of an individual marker on a cellular or molecular output. Studying multiple markers in different combinations is challenging to perform in prospective experiments, especially when limited by cell number. The index sorting technique allows retrospective subdivision, thereby permitting, in a single experiment, the analysis of high levels of one marker in combination with low levels of another and vice versa, which is particularly important when dealing with populations that are limited in number. This approach can be used to screen large numbers of possible combinations in individual experiments before proceeding—in the case of HSCs—to validation of self-renewal properties that would require long-term serial transplantation experiments.

Previous experiments have implicated CD150 intensity as a marker of lineage bias in HSCs, with high CD150 expression being associated with a relative underproduction of lymphoid cells [4,18,19]. These experiments required predefined gating strategies and, consequently, numerous transplantation experiments. Moreover, they restricted the questions to a single marker (CD150) to avoid having too many different experimental arms. Index sorting permits the study of multiple combinations at the same time that can be retrospectively analyzed, thereby greatly reducing the numbers of in vivo experiments required. In this study, we were thus able to illustrate the impact of another marker (EPCR) in combination with CD150 expression in many more combinations than could be tested with the available cell numbers.

Numerous techniques have been developed that could add further dimensionality to an index sorting experiment coupled with single-cell functional assays [20]. For example, in the stem cell in vitro experiments described here, one could add any number of additional markers to the E-SLAM protocol and, without changing the sorted cell population, be able to simultaneously assess the new marker's impact on cell proliferation, division kinetics, and differentiation capacities. Index sorting could also be used to improve purities in any fraction of cells for which candidate markers exist. Recent advances in flow cytometry permit assessment of 14–18 parameters, and tools such as spectral analysis promise even more possible combinations. Cellular systems that have detectable readouts at the single-cell level [20–22] can now use index sorting to couple these readouts to individual cell characteristics in a high-throughput and efficient manner.

## Author contributions

Experiments were designed by RS, NKW, and DGK with assistance from BG and CC. HSC isolation was performed by DGK, NKW, JCMP, and RS with assistance from CC and MM. Single-cell gene expression profiling was performed by NKW. Single-cell HSC assays and flow cytometry analysis were performed by DGK, JCMP, and RS; DGK. RS wrote the article with input from BG, CC, and NKW.
